# Simplified Model of PKCγ Signaling Dysregulation and Cytosol-to-Membrane Translocation Kinetics During Neurodegenerative Spinocerebellar Ataxia Type 14 (SCA14)

**DOI:** 10.3389/fnins.2019.01397

**Published:** 2020-01-31

**Authors:** Naveed Aslam, Farah Alvi

**Affiliations:** ^1^BioSystOmics, Bellaire, TX, United States; ^2^Department of Chemistry and Chemical Engineering, Lahore University of Management Sciences, Lahore, Pakistan; ^3^Department of Physics, COMSATS University Islamabad, Islamabad, Pakistan

**Keywords:** PKCγ translocation kinetics, dysregulated signaling, spinocerebellar ataxia, neurodegeneration, mutant

## Abstract

Spinocerebellar ataxia type 14 (SCA14) is an autosomal neurodegenerative disease clinically characterized by progressive ataxia in the patient’s gait, accompanied by slurred speech and abnormal eye movements. These symptoms are linked to the loss of Purkinje cells (PCs), which leads to cerebellar neurodegeneration. PC observations link the mutations in PRKCG gene encoding protein kinase C γ (PKCγ) to SCA14. Observations also show that the link between PKCγ and SCA14 relies on a gain-of-function mechanism, and, in fact, both positive and negative regulation of PKCγ expression and activity may result in changes in cellular number, size, and complexity of the dendritic arbors in PCs. Here, through a systems biology approach, we investigate a key question relating to this system: why is PKCγ membrane residence time reduced in SCA14 mutant PCs compared to wild-type (WT) PCs? In this study, we investigate this question through two contrasting PKCγ signaling models in PCs. The first model proposed in this study describes the mechanism through which PKCγ signaling activity may be regulated in WT PCs. In contrast, the second model explores how mutations in PKCγ signaling affect the state of SCA14 in PCs. Numerical simulations of both models show that, in response to extracellular stimuli-induced depolarization of the membrane compartment, PKCγ and diacylglycerol kinase γ (DGKγ) translocate to the membrane. Results from our computational approach indicate that, for the same set of parameters, PKCγ membrane residence time is shorter in the SCA14 mutant model compared to the WT model. These results show how PKCγ membrane residence time is regulated by diacylglycerol (DAG), causing translocated PKCγ to return to the cytosol as DAG levels drop. This study shows that, when the strength of the extracellular signal is held constant, the membrane lifetime of mutant PKCγ is reduced. This reduction is due to the presence of constitutively active mutant PKCγ in the cytosol. Cytosolic PKCγ, in turn, leads to phosphorylation and activation of DGKγ while it is still residing in the cytosol. This effect occurs even during the resting conditions. Thus, the SCA14 mutant model explains that, when both DAG effector molecules are active in the cytosol, their interactions in the membrane compartment are reduced, critically influencing PKCγ membrane residence time.

## Introduction

Clinically, the term “ataxia” describes abnormal limb movements and poor limb coordination (Shimobayashi, 2016). The most common form of ataxia is cerebellar ataxia, which can be linked to dysfunction either within the cerebellum or the cerebellar connecting pathways (Shimobayashi, 2016). Spinocerebellar ataxia (SCA) is a disease that manifests as dysfunction in the spinocerebellum, the part of the cerebellar cortex that receives somatosensory input from the spinal cord (Soong and Paulson, 2007; Carlson et al., 2009; Paulson, 2009). SCA14 is a rare form of SCA that can be inherited through autosomal dominance (Duenas et al., 2006; Shimobayashi, 2016). SCA14 has been linked to missense point mutations, deletions, or splice site mutations in the PRKCG coding region of PKCγ (Yamashita et al., 2000). Previous studies have linked almost 30 types of deletions or missense mutations in the PRKCG gene to SCA14-related symptoms (Shimobayashi, 2016). PKCγ is principally expressed in the central nervous system (CNS) and predominantly found in PCs (Saito et al., 1988; Saito and Shirai, 2002; Schrenk et al., 2002; Yabe et al., 2003). PKCγ is considered one of the key factors that control cerebellar development. SCA14 disease onset ranges from childhood to late adulthood, and usually does not result in a shorter lifespan. Generally, clinical symptoms of SCA14 include ataxia, dysarthria, oculomotor dysfunction, vertigo, facial myokymia, and myoclonus (Shimobayashi, 2016). Post-mortem neurohistological–pathological studies of patients with SCA14 have shown a pronounced reduction in the number of cerebellar Purkinje cells (PCs), as well a reduction in cellular size and complexity of the dendritic arbor (Brkanac et al., 2002).

An interesting possibility, supported by previous experimental observations, is that SCA14 might be linked to increases in PKCγ activity (Metzger and Kapfhammer, 2000; Adachi et al., 2008; Shimobayashi, 2016; Wong et al., 2018). Studies have shown that PMA-induced chronic PKCγ activation in cerebellar slice cultures drastically inhibits the growth and development of the PC dendritic tree (Metzger and Kapfhammer, 2000). This result could indicate that degeneration of the PC dendritic tree during SCA14 may be caused by increased PKCγ activity (Verbeek et al., 2005). Furthermore, 19 out of 20 spontaneous mutations found in the PKCγ gene of SCA14 patients showed increased constitutive PKCγ activity (Adachi et al., 2008). How constitutively active PKCγ may contribute to neurodegeneration is not clear. However, there is evidence that, despite an enhanced basal activity of the PKCγ isoform, there may be a deficit recruitment or regulation of downstream targets linked to a loss of specific cerebellar functions.

One possibility may be related to the membrane residence duration of PKCγ (Adachi et al., 2008; Shuvaev et al., 2011; Wong et al., 2018). In order to achieve precise regulation of PKCγs’ downstream targets, its membrane residence time must be exquisitely regulated (Adachi et al., 2008; Shuvaev et al., 2011; Shimobayashi, 2016). If the amount of time PKCγ spends in the membrane compartment is altered, this may lead to aberrant regulation and/or recruitment of downstream signaling molecules. Previous observations suggest that in PCs, the PKCγ membrane residence time is controlled by the amplitude and duration of DAG signaling. Experimental observation in Chinese hamster ovary (CHO) cells indicate that DAG signaling could contribute to a functional coupling of PKCγ and its regulator DGKγ (Yamaguchi et al., 2006; Goto and Kondo, 1999; Mérida et al., 2008). These results show (Yamaguchi et al., 2006) that in the membrane compartment, DGKγ regulates the activity of PKCγ through phosphorylating DAG, and thus inducing its metabolism. Our previous work elucidated the role of this functional coupling and showed how the timing of DGKγ and PKCγ colocalization in the membrane compartment is important for DAG signaling regulation in both CHO cells and PCs (Aslam and Alvi, 2017, 2019).

In addition, previous data indicate that, compared to wild-type, mutant PKCγ has higher basal level activity, but reduced membrane compartment residence time (Shuvaev et al., 2011). This may explain aberrant downstream signaling in mutant models of PKCγ. For example, one study revealed a decrease in PKCγ signaling, measured by canonical transient receptor potential channel (TRPC3) phosphorylation, when a PKCγ mutant remained at the membrane for significantly less time than wild-type PKCγ (Adachi et al., 2008). Altered PKCγ phosphorylation or recruitment may contribute to cerebellar dysfunction and apoptosis in PCs.

The purinergic receptors belong to a class of plasma membrane molecules that have been critically implicated in the regulation of physiological and pathological responses such as learning memory, inflammation, motor control, and sleep. They are classified into P_1_ (adenosine-activated) and P_2_ (ATP-activated) subfamilies. Among the P_2_ subfamily, the P_2_X subgroup is ionotropic and the P_2_Y subgroup is metabotropic nucleotide receptor. The P_2_Y subfamily has eight members and are expressed in cells of the nervous system. Among this subgroup, P_2_Y_1_, P_2_Y_2_, P_2_Y_4_, P_2_Y_6_, and P_2_Y_11_ receptors are coupled to G_q_-protein (Weisman et al., 2012; Guzman and Gerevich, 2016). The activation of this group by general currency in energy conversion, i.e., adenosine triphosphate (ATP) or nucleotide, uridine-5′-triphosphate (UTP), induces the G_q_-dependent activation of phospholipase C (PLC), which promotes the hydrolysis of plasma membrane phospholipid phosphatidylinositol 4,5, bisphosphate (PIP_2_) to generate second messenger DAG and inositol 1,4,5 triphosphate (IP_3_). Both these second messengers are critical for the release of intracellular Ca^+2^ from stores, exchange of Ca^+2^ with extracellular pools, and activation of PKCγ and DGKγ.

This work explores why PKCγ membrane residence time is reduced in PCs in the mutant model, despite higher kinase activity. The following experimental observations provide the basis for this study: (1) Depolarization-induced activation of mGluR1 pathways leads to the membrane translocation of both mutant and wild-type PKCγ (Shuvaev et al., 2011; Shimobayashi, 2016). (2) Mutant PKCγ is constitutively active (Adachi et al., 2008; Shuvaev et al., 2011; Shimobayashi, 2016). (3) During a depolarization-induced activation event, the wild-type PKCγ membrane residence time is 19 s. The mutant PKCγ membrane residence time is 6 s (Shuvaev et al., 2011). (4) Depolarization-induced stimulation of both mutant and wild-type PKCγ results in rapid membrane translocation followed by a slow return to the cytosol (Adachi et al., 2008; Shuvaev et al., 2011; Shimobayashi, 2016). (5) In response to ATP stimulation, PKCγ and DGKγ form a subtype-specific functional coupling, which regulates DAG signaling in the membrane compartment in the CHO cell system (Yamaguchi et al., 2006). (6) Observations in post-mortem SCA14 cerebellum and human patient-derived induced pluripotent stem cells (iPSCs) show that, when activated, wild-type PKCγ is distributed in the membrane and cytosolic compartment, whereas mutant PKCγ tends to localize in cytosol (Wong et al., 2018), thus indicating that mutant PKCγ is impaired in its ability to translocate, or be retained at, the plasma membrane. (7) Additional observations from SCA14 iPSCs and SCA14 PCs indicate that mutant PKCγ present in cytosol is in hyperactive state (Wong et al., 2018). (8) Moreover, this molecule leads to a robust increase in PKCγ substrate phosphorylations as observed by increase in PKCγ substrate antibody and its well-established target, i.e., myristoylated alanine-rich C-kinase substrate (MARCKS) in SCA14 cerebellum (Wong et al., 2018). In this work, we test the hypothesis that due to reduced local signaling, the lifetime of mutant PKCγ is reduced in the membrane compartment.

This work tests this hypothesis by constructing two contrasting models (WT and mutant). The simulations mimicking mutant model includes a constitutively active PKCγ in the cytosol (Adachi et al., 2008; Shuvaev et al., 2011; Shimobayashi, 2016; Wong et al., 2018), which, in turn, phosphorylates and activates DGKγ even during basal conditions. The simulations representing the wild-type model includes inactive and dormant PKCγ and DGKγ in the cytosol during basal conditions and models translocation to the membrane compartment upon stimulation. The wild-type model shows membrane activation of DGKγ in a stimulation-dependent manner, whereas the mutant model relies on stimulation-independent cytosolic DGKγ activation. Both models are numerically perturbed by the same stimulation strength levels. For both models, all kinetic rate constants and translocation parameters are set at the identical numerical values. The overall goal of this study is to compare the effects on PKCγ membrane residence duration in the mutant and wild-type models through numerical experimentation, with all other parameters held constant. Here, we show that, despite higher activity levels membrane residence, duration for PKCγ in the mutant model is three times shorter than the wild-type. We also show that, when a stimulation pulse is applied to mimic depolarization-induced activation of mGluR1, PKCγ translocation from the cytosol to the membrane is induced. The manner and time scale of this induction is consistent with experimental observations (Shuvaev et al., 2011). Depolarization-induced local DAG generation in the membrane compartment also induces DGKγ translocation from the cytosol to the membrane, where the molecule acts as a key negative regulator of DAG levels in the membrane compartment.

Experimental results suggest that despite the constitutively active nature of mutant molecule, the impaired translocation and shorter membrane residence time result in lower PKCγ concentrations in the membrane compartment of PCs (Shuvaev et al., 2011; Wong et al., 2018). This observation could be linked to the association of mutant PKCγ with large EPSC amplitudes in PCs (Shuvaev et al., 2011). Our simulations are consistent with these observations, as the maximum membrane-to-cytosol (M/C) ratio in the mutant model is half of the ratio in the wild-type model. This indicates that reductions in PKCγ concentration in the membrane compartment may be linked to impaired PC functionality.

## Materials and Methods

### Biochemical Reactions

**FIGURE 1:**
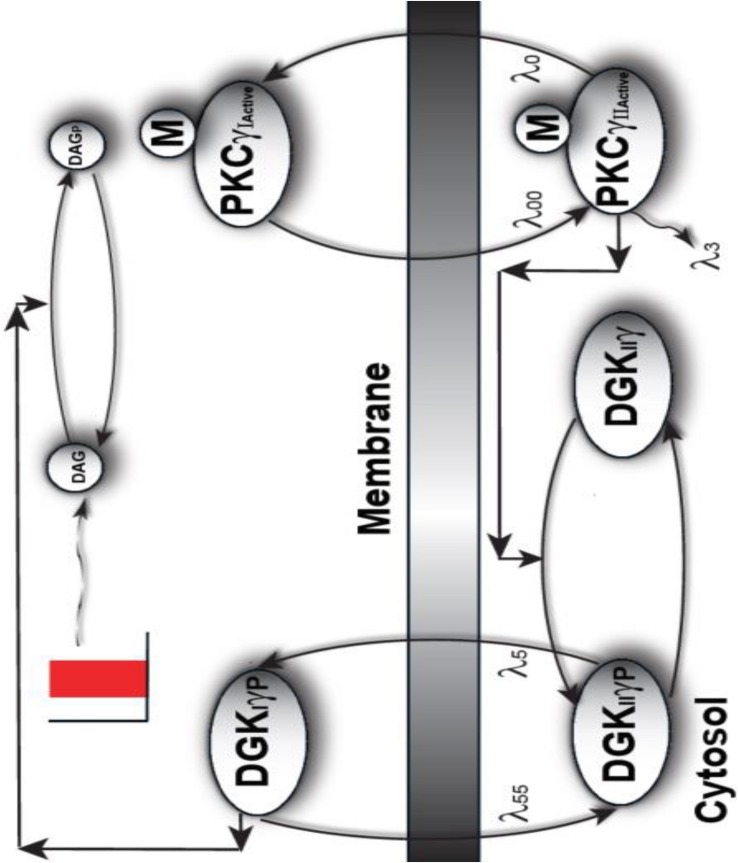
A proposed regulatory model of mutant PKCγ, translocation in the SCA14-associated Purkinje cells of the cerebellum. This is a two-compartment model where one compartment is cytosol, whereas the other compartment is plasma membrane. This model provides the mechanistic basis of how the translocation of mutant PKCγ and DGKγ molecules could be regulated in SCA14 disease. This model suggests that in diseases associated with cPCs, the mutant PKCγ is constitutively active and resides in the cytosol. In turn, this constitutively active molecule induces the phosphorylation and activation of the cytosolic DGKγ molecule even during the basal or unstimulated conditions. In contrast, to the wild-type model, the current model suggests that both PKCγ and DGKγ are active and cytosolic even during basal conditions. Depolarization-induced activation of purinergic receptor leads to the local generation of DAG and, in turn, induces the translocation of both mutant PKCγ and DGKγ from cytosol to membrane. Once in the plasma membrane compartment, the already active DGKγ molecule directly converts DAG to PA through DAG phosphorylation.

The following model describes a mutant PKCγ signaling model in PCs. This simulation models mutant and active PKCγ and DGKγ molecules. These biochemical interactions describe how local DAG generation leads to a signaling loop between PKCγ and DGKγ in our mutant models of PCs. The simulated interactions of molecules within the PKCγ and DKCγ loop (Figure 1) are based on standard Michaelis–Menten kinetics. The following sets of biochemical reactions are used to describe the molecular interactions that occur within the PKCγ and DKCγ loop. The dynamic variables are DAG, DGKγ, and PKCγ. Subscript I represents the concentration in the first compartment, the plasma membrane. Subscript II denotes the concentration in the second compartment, which represents the cytosol. The superscript Active represents the activated form of a molecule and the subscript P represents the phosphorylated form of a molecule. The subscript Mutant represents the mutant form of a molecule. The phosphatase P is approximated as a fixed parameter. The parameter S1 denotes purinergic receptor stimulation, which leads to the rapid generation of DAG molecules.

(1) $${\text{PKC}}^{\text{Active}}{}_{\gamma \text{II.Mutant}}\;\overset{{\text{λ}}_{\text{0}}}{\to }{\text{PKC}}^{\text{Active}}{}_{\gamma \text{IMutant}}$$

(2) $${\text{PKC}}^{\text{Active}}{}_{\gamma \text{IMutant}}\;\overset{{\text{λ}}_{\text{00}}}{\to }{\text{PKC}}^{\text{Active}}{}_{\gamma \text{II Mutant}}$$

(3) $${\text{DGK}}_{\gamma \text{IIP}}\overset{\lambda_{5}}{\to }\;{\text{DGK}}_{\gamma \text{IP}}$$

(4) $${\text{DGK}}_{\gamma \text{IP}}\overset{\lambda_{55}}{\to }\;{\text{DGK}}_{\gamma \text{IIP}}$$

(5) $$S_{1}\overset{k_{1}}{⟶}\text{DAG}$$

(6) $$\begin{array}{l} {\text{DGK}}_{\gamma \text{II}}{\text{+PKC}}^{\text{Active}}{}_{\gamma \text{IIMutant}} \end{array}\begin{array}{l} {}_{\underset{{\text{K}}_{3}}{\leftarrow }}^{\overset{{\text{K}}_{2}}{\to }}{\text{C}}_{\text{1}}\overset{{\text{K}}_{\text{4}}}{\to }{\text{DGK}}_{\gamma \text{IP}}{\text{+PKC}}^{\text{Active}}{}_{\gamma \text{IIMutant}} \end{array}$$

(7) $${\text{DGK}}_{\text{γIIp}}\text{+P}\overset{{\text{k}}_{\text{5}}}{\to }{\text{DGK}}_{\text{γII}}\text{+P}$$

(8) $${\text{DAG+DGK}}_{\gamma \text{IP}}{\;}_{\underset{{\text{K}}_{7}}{\leftarrow }}^{\overset{{\text{K}}_{6}}{\to }}{\text{C}}_{2}\;\overset{{\text{K}}_{8}}{\to }\;{\text{DGK}}_{\gamma \text{IP}}+{\text{DAG}}_{\text{P}}$$

(9) $${\mathrm{DAG}}_{P}\overset{k_{g}}{⟶}P.A$$

(10) $${\text{PKC}}_{\text{II}\gamma }{}^{\text{Active}}\text{mutant}\overset{\lambda_{3}}{\to }\;\left[\;\right]$$

The signaling described in the above model starts during basal conditions. The constitutively active cytosolic mutant PKCγ molecule leads to phosphorylation and activation of DGKγ in the cytosol. This event is described by Equation (6). A pulse stimulation that mimics depolarization-induced stimulation of the pathway leads to DAG generation at the plasma membrane. This local event, causing generation of a second messenger, is described in Equation (5). DAG generation then stimulates the migration of dormant and active mutant PKCγ_II_ and DGKγ_II_ from the cytosol to the plasma membrane. These migration events are described by Equations (1) and (3). Here, the migration rates “λ_0_” and “λ_5_” are described through functions that are directly proportional to DAG concentrations, but with different slopes. Then, the active γ-molecules in the plasma membrane compartment re-translocate to the cytosol with fixed migration rates, “λ_00_” and “λ_55_,” as described by Equations (2) and (4). Once at the plasma membrane, DGK*γ*_I_ causes DAG metabolism through its phosphorylation, as described by Equation (8). Phosphorylated DAG is converted to phosphatidic acid (PA) as shown in Equation (9). PA is another key signaling lipid that may function directly as a key regulatory molecule. The dephosphorylation event of DGKII*γ*_P_ is described by Equation (7).

### Induction

During simulations, the membrane depolarization-induced activation of purinergic receptor is mimicked through the application of a brief pulse. The local biosynthesis of second messenger DAG is modulated through a 1.0-min pulse stimulation. DAG generation in the membrane compartment induces the translocation and activation of its effector molecules.

### Temporal Dynamics

The differential equations resulting from the above interactions [Equations (1) to (9)] were integrated through nonlinear solvers using MATLAB (MathWorks). The dynamical coefficients’ values were estimated from limited experimental data (Shuvaev et al., 2011). Unknown rate constants were scaled to obtain dynamics that were comparable to experimental values (Shuvaev et al., 2011). Unless otherwise stated, all of the molecular concentrations in the model are expressed as pg/ml and time is represented in seconds.

## Results

### Comparative Models Describing DAG Signaling in Mutant and Wild-Type PCs

The DAG signaling model we propose for mutant PCs (Figure 1) in cerebellar PCs is composed of two molecular components. The first component is mutant PKCγ, which can be active and cytosolic (PKC^Active^γ_II-Mutant_) or active and membrane (PKC^Active^γ_I-Mutant_). The second component is DGKγ, which can be cytosolic and inactive (DGK_II_γ); cytosolic, active, and phosphorylated (DGK${}_{\text{II}}\,\gamma_{\text{P}}^{\text{A}}$); or active and phosphorylated in the membrane compartment (DGKIγAP). In the mutant signaling model, PKCγ is constitutively active and leads to phosphorylation and activation of DGKγ in the cytosolic compartment. This occurs even during basal conditions. In this model, depolarization-induced stimulation leads to local DAG generation in the membrane compartment. This leads to cytosol-to-membrane translocation of active PKCγ and DGKγ. Once in the membrane compartment, already active and phosphorylated DGKγ stimulates DAG metabolism. DAG levels are quickly reduced by the molecule’s conversion to PA. Once DAG levels in the membrane drop, both molecules relocate back to the cytosol.

**FIGURE 2:**
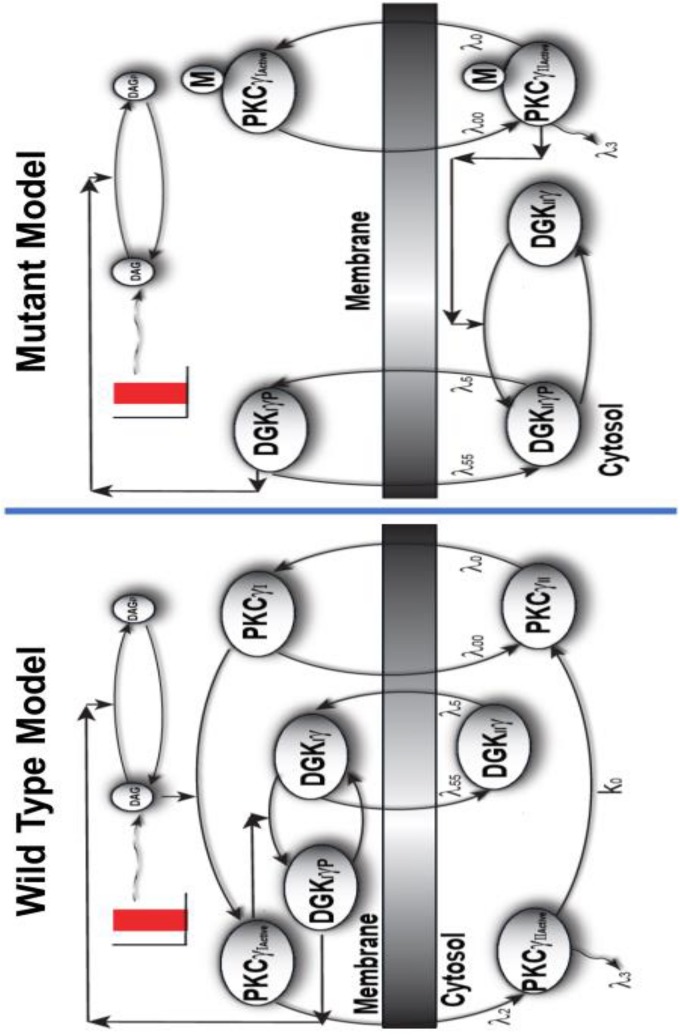
The comparison of two-compartment, wild-type, and mutant models of local DAG signaling in PCs. Depolarization-induced local generation of DAG in PCs, in turn, leads to the functional coupling between PKCγ and DGKγ molecules. The wild-type model is characterized by dormant and inactive PKCγ molecule residing in cytosol, whereas the mutant model is characterized by active PKCγ isoform in the cytosol. In the wild-type model, the depolarization-induced activation of purinergic receptor and local generation of DAG stimulates the translocation of PKCγ and DGKγ molecules from cytosol to membrane compartments. Once in the membrane compartment, the DAG binds with PKCγ and activates it, which, in turn, activates DGKγ through phosphorylation. In the membrane compartment, the phosphorylated and active DGKγ molecule stimulates the DAG metabolism, thus restricting its own activation. Once DAG is converted to PA in membrane compartment, both these molecules return to their dormant forms in cytosol. In contrast, the signaling in the mutant model is reduced as in the mutant model of PCs; the PKCγ is in constitutively active state and leads to the phosphorylation and activation of DGKγ in the cytosol even during unstimulated conditions. On stimulation, as DAG is generated in the membrane compartment, both these molecules migrate to membrane where the already activated DGKγ molecule converts DAG to PA. It seems that signaling in the mutant model is reduced due to the constitutively active form of PKCγ in cytosol.

In contrast, Figure 2 shows that PKCγ can reside in one of four states in the wild-type model (Supplementary Material S2: Biochemical Reactions describing fast kinetics wild-type model). The four states are cytosolic dormant (PKCγ_II_), inactive membrane (PKCγ_I_), active membrane (PKCγAI), and active cytosolic (PKCγAII). In the mutant cascade, PKCγ exists in only two distinct forms. This is because PKCγ is inactive in the wild-type model in its dormant cytosolic state, but constitutively active in the mutant model while residing in the cytosol. This difference leads to a reduced PKCγ cycle in the mutant model (Figures 1, 2). Interestingly, cytosolic DGKγ in the wild-type model is inactive, and only phosphorylated and activated in the membrane compartment. In contrast, in the mutant model, it is activated and phosphorylated in the cytosol by constitutively active PKCγ. The two-state mutant model and four-state wild-type model of PKCγ (Figure 2) are only simplistic descriptions of a complex PKCγ cycling process. A more realistic, complex PKCγ cycling model should account for molecular events like translocation, binding, activation, and re-translocation of active PKCγ to the cytosol. In addition, it should include deactivation of PKCγ back to its dormant form in the cytosol. While the processes modeled in this manuscript are complex, this study approximates the processes using simpler biochemical kinetic events that preserve key qualitative features. Cytosol-to-membrane translocation of both PKCγ and DGKγ in these mutant and wild-type simulations is described through proportionality functions of DAG concentrations. In addition, this study assumes non-negligible basal levels of PKC^Active^γ_*II*-Mutant_ (100 pg/ml) and DGK_II_γ (100 pg/ml) in the mutant and PKCγ_II_ (100 pg/ml) and DGK_II_γ (100 pg/ml) in the wild-type model. In contrast, the basal concentration of all other forms of PKCγ and DGKγ is negligible. The biochemical reaction and translocation rates of this molecular loop were obtained by fitting the depolarization-induced temporal dynamics of PKCγ translocation and re-translocation obtained from PCs in cerebellar slices as described in previously published studies (Shuvaev et al., 2011).

In both the mutant and wild-type models, DGKγ inhibits its own translocation and that of PKCγ by converting DAG into PA at the plasma membrane. Our models show that depolarization-induced stimulation in the membrane compartment leads to DAG generation. DAG generation, in turn, stimulates the DGKγ and PKCγ translocation. In the mutant model, once the already active DGKγ is at the plasma membrane, it directly induces DAG metabolism. In contrast, in the wild-type model, DGKγ undergoes an activation event before becoming competent to metabolize DAG. These events restore DAG homeostasis, thus reducing migration to the membrane compartment. In both these models, local DAG generation is counterbalanced by DGKγ-assisted metabolism in the membrane compartment.

Regulation of depolarization-induced activation of G protein-coupled receptor (GPCR) pathways is a complex process (Shuvaev et al., 2011). GPCR agonist mGluR1 stimulates PLC-mediated hydrolysis of phosphatidylinositol 4,5-biphosphate to produce inositol triphosphate (IP_3_) and DAG (Shuvaev et al., 2011). In this study, although we are primarily focusing on the downstream signaling of the purinergic receptor pathway, we modeled local DAG biosynthesis in both the mutant and wild-type models by using a brief pulse of variable intensity. This pulse is used to mimic the effects of GPCR agonist mGluR1. This approach is simple in that it ignores the details of purinergic receptor-induced DAG biosynthesis. Since our primary focus is regulation of DAG homeostasis, and not DAG biosynthesis, this study employs this simple approach.

### Comparison of PKCγ and DGKγ Translocation Characteristics in Mutant and Wild-Type PC Models Through Numerical Simulations

**FIGURE 3:**
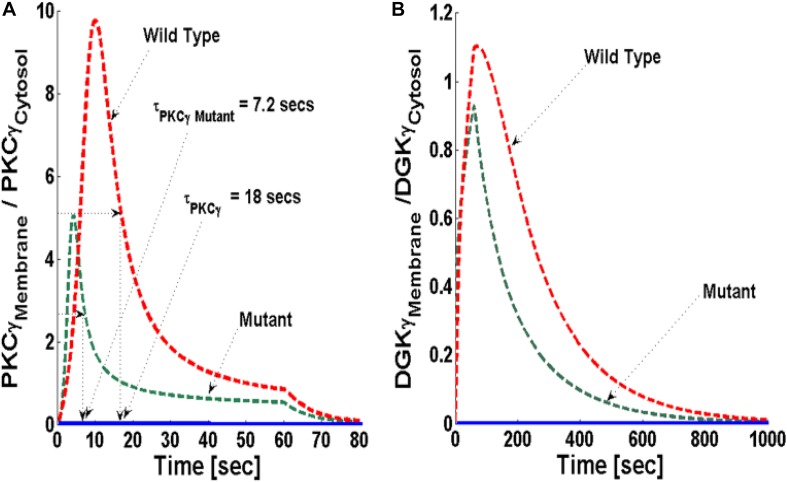
The simulations mimicking the comparison of depolarization-induced translocation of PKCγ and DGKγ molecules in the mutant and wild-type models of PCs. These results show the membrane-to-cytosol (M/C) ratio of PKCγ and DGKγ molecules in response to a brief 1-min pulse, which leads to the rapid generation of DAG in the membrane compartment. Here, the strength of stimulation is controlled by setting the pulse parameter “S_1_” at 20. The generation of the second messenger, in turn, stimulates the translocation of both PKCγ and DGKγ from cytosol to membrane. Here, the solid line represents the non-stimulation and the dashed line represents the stimulation condition (green dashed line, mutant; red dashed line, wild-type PCs). **(A)** The translocation characteristics of the PKCγ molecule in both mutant and wild-type models. These results suggest that for identical strength and duration of stimulation, the cytosol-to-membrane migration kinetics of PKCγ molecule are much faster in mutant models compared to wild-types. Compared to wild-types, the membrane residence time of PKCγ molecule is shorter in mutant models, i.e., 7.2 s for mutant and 18 s for wild-type models. **(B)** The translocation characteristics of the DGKγ molecule in both mutant and wild-type models. These results show that the membrane residence time of the DGKγ molecule is shorter in mutant models compared to models representing wild-type PCs.

Next, the translocation characteristics of PKCγ and DGKγ in the mutant and wild-type models were compared through numerical simulations. Translocation characteristics were determined by measuring two key properties. First, translocation kinetics from cytosol to membrane were measured. Next, the membrane-to-cytosol (M/C) ratio was measured. The kinetics of translocation are determined by measuring the time that both DAG effector molecules spend in the membrane compartment. During the course of simulations, the M/C ratio is measured by computing the total amount of DAG effector molecules in the membrane and cytosolic compartment at every time step and then taking the ratio. In simulations mimicking the post depolarization-induced receptor activation, the speed of translocation response is described using kinetic parameters. In contrast, during these numerical experiments, the intensity of this response is described using the M/C ratio of PKCγ and DGKγ in the membrane and cytosolic compartments. In simulations mimicking both the mutant and wild-type models, the mGluR1-induced activation of GPCR and subsequent DAG generation were implemented using a brief 1-min pulse, as described in the previous section. The strength of pulse is described by an arbitrary parameter S_1_. S_1_ was set at an arbitrary level of 20. In the absence of a pulse (Figure 3, solid blue lines), there is no *de novo* DAG biosynthesis and the system is fixed in its basal state. In the basal state, both molecules reside in the cytosol with no possibility of translocation.

The stimulation-induced temporal dynamics of PKCγ in the mutant (Figure 3A, dashed green line) and wild-type (Figure 3A, dashed red line) models show two phases of translocation. The first is an early phase, in which PKCγ migrates from the cytosol to the membrane. The second phase that follows is a resolution phase in which PKCγ relocates back to the cytosol. These results show that, for the same level of stimulation pulse, the translocation of PKCγ from the cytosol to the membrane is faster in the mutant model compared to wild-type. In contrast, the translocation intensity, measured by the M/C ratio of PKCγ, is lower in the mutant model compared to wild-type (Figure 3A, dashed green and red line). These results show that cytosolic-to-membrane molecular migration in both models is dependent on local DAG concentration at the plasma membrane and is linked to *de novo* DAG biosynthesis. In both the mutant and wild-type models, DAG concentration can be controlled through the duration and amplitude of pulse stimulation at the plasma membrane compartment.

**FIGURE 4:**
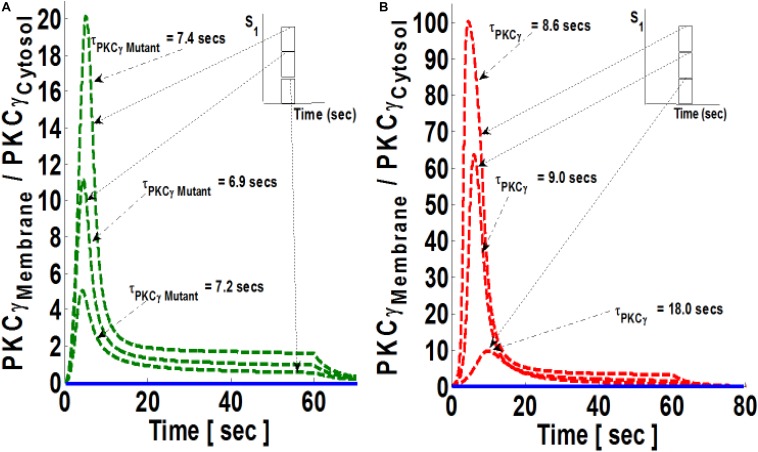
The simulations mimicking the effect of stimulation strength on the comparative translocation kinetics of PKCγ isoform in the mutant and wild-type models of PCs. Here, the strength of stimulation is controlled by setting the pulse parameter “S_1_” at different levels. The parameter S_1_ is set at arbitrary values of 20, 40, and 60 (inset). The duration of pulse for all these three cases is 1 min. Here, the solid line represents the non-stimulation and the dashed line represents the stimulation condition (green dashed line, mutant; red dashed line, wild-type PCs). **(A)** Translocation characteristics of PKCγ in the mutant model. Here, results show that maximum levels of M/C ratio of PKCγ increases with increase in the strength of parameter “S_1_”; however, the residence time of mutant PKCγ first decreases and then increases with pulse strength (S_1_ = 20, leads to maximum M/C levels of 5 and τ_PKCγmutant_ = 7.2 s; S_1_ = 40, leads to maximum M/C levels of 11 and τ_PKCγmutant_ = 6.9 s; S_1_ = 60, leads to maximum M/C levels of 20 and τ_PKCγmutant_ = 7.4 s). **(B)** Translocation characteristics of PKCγ in the wild-type models. Here, results show that maximum levels of the M/C ratio of PKCγ increases with increase in the strength of parameter “S_1_,” and the residence time of wild-type PKCγ decreases with pulse strength (S_1_ = 20, leads to maximum M/C levels of 10 and τ_PKCγ_ = 18 s; S_1_ = 40, leads to maximum M/C levels of 64 and τ_PKCγ_ = 9 s; S_1_ = 60, leads to maximum M/C levels of 100 and τ_PKCγ_ = 8.6 s).

In both models, the PKCγ translocation rate is a directly proportional function of DAG concentration (Supplementary Material S1: Table S1 and Supplementary Material S3: Table S2). When the mutant model was perturbed with different levels of stimulation, the results indicate that when stimulation strength is set at moderate levels (Figure 4A, inset; pulse strength parameter S_1_ = 20), only a small pool of DAG is generated at the plasma membrane. This induces a low-intensity migration event reflected by a maximal M/C ratio. The M/C ratio is linked to pulse strength (Figure 4A). Higher levels of stimulation generate (Figure 4A, inset; pulse strength parameter S_1_ = 40 and 60) much larger pools of DAG at the plasma membrane. This induces migration of a large pool of PKCγ to the membrane. Interestingly, perturbation of the wild-type model (Figure 4B) also generates the same pattern with regard to the M/C ratio of PKCγ. However, at higher stimulation strengths, the maximal M/C ratio of PKCγ in the wild-type model is five-times more than the ratio at moderate stimulation. In contrast, it is 10-times more at even higher stimulation strength, showing the effects of DAG generation on translocation of PKCγ from the cytosol to the membrane.

In this study, DGKγ translocation is spatially similar, but temporally distinct, from PKCγ. The migration of DGKγ is also controlled by DAG concentrations at the plasma membrane. The sensitivity of this migratory event is different from that of PKCγ. DGKγ translocation is also described by a proportionality function (Supplementary Material S1: Table S1 and Supplementary Material S3: Table S2) of DAG concentration at the plasma membrane. Compared to PKCγ, however, the slope of this function is much smaller. This means that DGKγ migration from the cytosol to the plasma membrane requires a much larger increase in DAG concentration at the plasma membrane. This approach was adopted so that the proposed models align with previous experimental observations showing different translocation sensitivities of PKCγ and DGKγ to Ca^+2^ concentrations (Yamaguchi et al., 2006). These observations suggest that both PKCγ and DGKγ possess DAG-sensitive C_1_ and Ca^+2^-sensitive C_2_ domain. Interestingly, nanomolar elevation in Ca^+2^ concentration was enough to warrant PKCγ translocation, whereas DGKγ translocation required micromolar increase in Ca^+2^concentration (Yamaguchi et al., 2006).

At the plasma membrane, the coupling between PKCγ and DGKγ can be quantified by measuring colocalization time between these two molecules. Colocalization time is defined as the duration for which these molecules may interact with each other. This time interval also determines the duration during which a negative feedback loop is functional and effectively facilitating DAG metabolism. Colocalization time depends on DAG concentration in the plasma membrane, re-translocation rates of the inactive molecules, the remigration rate of active PKCγ, the activation rate and DAG binding activity of PKCγ, and activation and phosphorylation of DGKγ.

In order to estimate the parameters of proposed PKCγ and DGKγ interaction in PCs, we fixed the membrane residence duration of PKCγ in both mutant and wild-type models well within the experimentally reported ranges (Shuvaev et al., 2011) and determined the unknown parameters of molecular loop as well as the translocation and remigration rates of DGKγ. We also assumed that, similar to the CHO cell model, the sensitivity of PKCγ translocation to DAG concentration is higher than DGKγ (Supplementary Material S1: Table S1 and Supplementary Material S3: Table S2). We calibrated our models by matching the ranges of membrane residence duration of PKCγ in mutant and wild-type models of cPCs (Shuvaev et al., 2011). This ad hoc approach could be questioned, but in the absence of direct translocation data of DGKγ migration and remigration in PCs, we believe that this is a reasonable approximation. The assumption made here should be tested directly and the translocation rate of DGKγ should be measured directly in PCs. However, it is unlikely that the overall structure of this model and the conclusion drawn from this model will be significantly different based on these assumptions.

### Simulations Mimicking the Effect of the Rate of DAG Phosphorylation on the Translocation Characteristics of PKCγ in Mutant and Wild-Type Models of PCs

**FIGURE 5:**
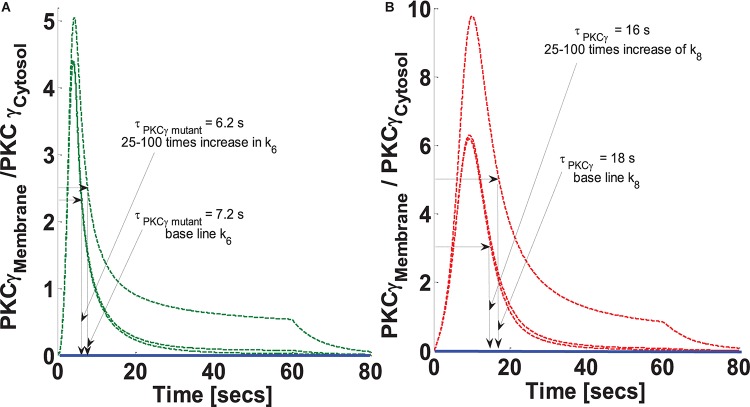
The simulations mimicking the effect of feed forward rate constant of DAG phosphorylation by DGKγ_P_ on the comparative translocation kinetics of PKCγ molecule in the mutant and wild-type models of PCs. Parameter k_6_ represent this rate constant in mutant models, whereas in wild-types, it is represented by k_8_. Here, the strength of stimulation is controlled by setting the pulse parameter “S_1_” at 20. The duration of pulse is set for 1 min. Here, the solid line represents the non-stimulation and the dashed line represents the stimulation condition (green dashed line, mutant; red dashed line, wild-type PCs). **(A)** Translocation characteristics of PKCγ in the mutant model. Here, results show that increasing the parameter k_6_ in the mutant model reduces the membrane residence time of PKCγ (at baseline, k_6_, τ_PKCγ mutant_ = 7.2 s; and 25–100 times increase in k_6_ leads to τ_PKCγmutant_ = 6.2 s). **(B)** Translocation characteristics of PKCγ in the wild-type models. Here, results show that increasing the parameter k_8_ in the wild-type model reduces the membrane residence time of PKCγ (at baseline, k_8_, τ_PKCγ_ = 18 s; and 25–100 times increase in k_8_ leads to τ_PKCγ_ = 16 s).

The residence time of PKCγ at the membrane is modulated by DAG levels. DAG metabolism at the membrane is regulated by active and phosphorylated DGKγ. DGKγ leads to DAG phosphorylation, an essential step for the conversion of DAG to PA. It is possible that the molecular events involved in DAG phosphorylation may influence the residence time of PKCγ at the membrane. This study addresses the question of how the DAG phosphorylation rate, “k_6_” in the mutant model and “k_8_” in the wild-type model, might influence the intensity and translocation of PKCγ and DGKγ from the cytosol to the membrane. The parameter “k_6_” in the mutant and “k_8_” in the wild-type model describe the feed forward rate constant of DAG phosphorylation by DGKγ_P_. This study tested the hypothesis that how an increase in parameters k_6_ and k_8_ could influence the translocation as well as membrane residence duration of PKCγ. The simulations compared three difference cases: (a) baseline k_6_ and k_8_ (k_6_ = 0.95 pM^–1^ s^–1^ and k_8_ = 0.95 pM^–1^ s^–1^); (b) 25-times increase in the k_6_ and k_8_; (c) 100-times increase in the k_6_ and k_8_. The results show that increasing the parameter k_6_ not only reduces the intensity of translocation but also the membrane residence time of PKCγ in the mutant model (Figure 5A). A 25-fold increase in k_6_ causes the maximum M/C ratio to decrease 10%. In contrast, membrane residence time decreased 13% in the mutant model (Figure 5A). Interestingly, increasing k_6_ from 25- to 100-fold had no effect on translocation or membrane residence duration for PKCγ in the mutant model (Figure 5A). The results from the wild-type model show that a 25-fold increase in k_8_ reduces PKCγ translocation intensity to almost 40%. In contrast, membrane residence time is reduced by only 11% (Figure 5B). Furthermore, an increase in k_8_ from 25- to 100-fold had an almost negligible influence on membrane residence duration and the magnitude of translocation (Figure 5B).

**FIGURE 6:**
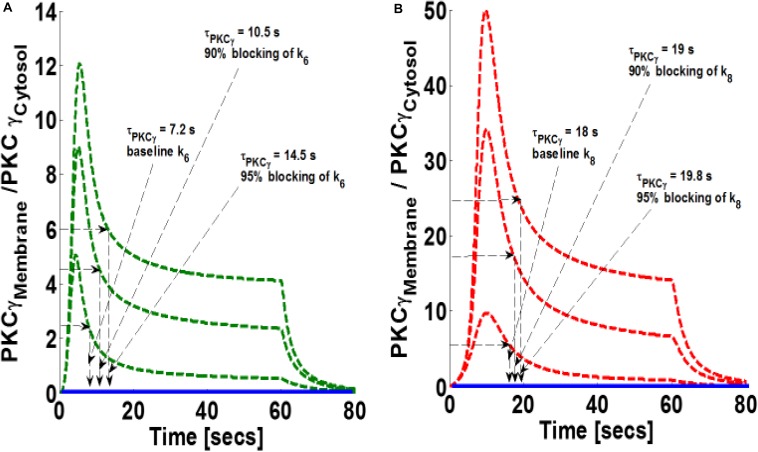
The simulations mimicking the effect of blocking of forward rate constant of DAG phosphorylation by DGKγ_P_ on the comparative translocation kinetics of PKCγ molecule in the mutant and wild-type models of PCs. Parameter k_6_ represent this rate constant in mutant models, whereas in wild-types, it is represented by k_8_. Here, the strength of stimulation is controlled by setting the pulse parameter “S_1_” at 20. The duration of pulse is set for 1 min. Here, the solid line represents the non-stimulation and the dashed line represents the stimulation condition (green dashed line, mutant; red dashed line, wild-type PCs). **(A)** Translocation characteristics of PKCγ in the mutant model. Here, results show that blocking the parameter k_6_ in the mutant model increases the membrane residence time of PKCγ (baseline, k_6_, τ_PKCγ mutant_ = 7.2 s; 90% blocking of k_6_ leads to τ_PKCγ mutant_ = 10.5 s; 95% blocking of k_6_ leads to τ_PKCγ mutant_ = 14.5 s). **(B)** Translocation characteristics of PKCγ in the wild-type models. Here, results show that blocking the parameter k_8_ in the wild-type model enhances the membrane residence time of PKCγ (baseline, k_8_, τ_PKCγ_ = 18 s; 90% blocking leads to τ_PKCγ_ = 19 s; 95% blocking leads to τ_PKCγ_ = 19.8 s).

Next, this study investigated how blocking DAG phosphorylation influences the magnitude of PKCγ translocation as well as PKCγ membrane residence duration in both the mutant and wild-type models. Our results indicate that blocking parameter k_6_ in the mutant model increases both the magnitude and membrane residence duration of PKCγ (Figure 6). Our results show that 90 and 95% blocking of k_6_ in the mutant model leads to maximum M/C ratios of 8.8 and 12.2, respectively. In contrast, PKCγ membrane residence duration times are 10.5 and 14.5 s, respectively (Figure 6A). These results indicate that 95% blocking in the mutant model leads to an almost twofold increase in the magnitude of translocation and membrane residence duration of PKCγ (Figure 6A). Similarly, our results indicate that blocking the DAG phosphorylation parameter, k_8_, in wild-type models also increases translocation intensity and membrane residence duration (Figure 6B). Our results show that 90 and 95% blocking of k_8_ in the wild-type model leads to 3 and 5-fold increases in the magnitude of translocation (Figure 6B; measured as maximum in M/C ratio). In contrast, only 5 and 10% increases in membrane residence duration were observed for the wild-type model of PKCγ in cPCs (Figure 6B).

### Simulations Mimicking the Influence of the DGKγ Activation Rate on the Translocation Characteristics of PKCγ

**FIGURE 7:**
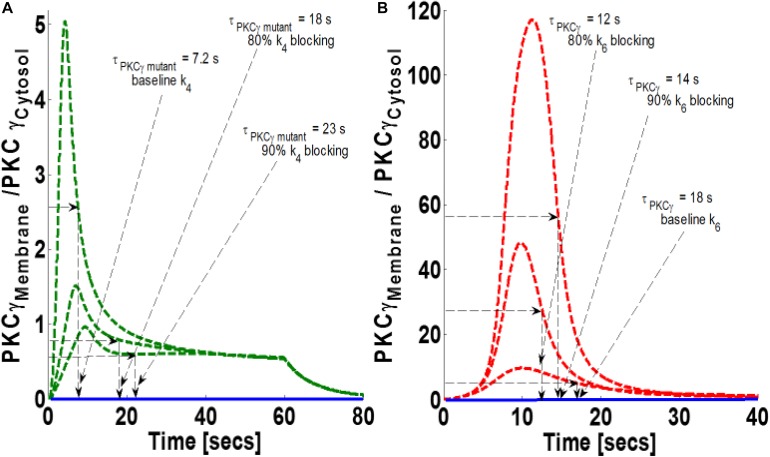
The simulations mimicking the effect of blocking the rate constant of DGKγ phosphorylation to DGKγ_P_ on the comparative translocation kinetics of PKCγ molecule in the mutant and wild-type models of PCs. Parameter k_4_ represents this rate constant in mutant models, whereas in wild-types, it is represented by k_6_. Here, the strength of stimulation is controlled by setting the pulse parameter “S_1_” at 20. The duration of pulse is set for 1 min; the solid line represents the non-stimulation and the dashed line represents the stimulation condition (green dashed line, mutant; red dashed line, wild-type PCs). **(A)** Translocation characteristics of PKCγ in the mutant model. Here, results show that blocking the parameter k_4_ in the mutant model increases the membrane residence time of PKCγ (baseline, k_4_, τ_PKCγ mutant_ = 7.2 s; 80% blocking of k_4_ leads to τ_PKCγ mutant_ = 18 s; 90% blocking of k_4_ leads to τ_PKCγ mutant_ = 23 s). **(B)** Translocation characteristics of PKCγ in the wild-type models. Here, results show that blocking the parameter k_8_ in the wild-type model reduces the membrane residence time of PKCγ (baseline, k_6_, τ_PKCγ_ = 18 s; 80% blocking leads to τ_PKCγ_ = 12 s; 90% blocking leads to τ_PKCγ_ = 14 s).

DGKγ phosphorylation and activation play key roles in regulating DAG homeostasis in the mutant and wild-type models. In the mutant model, DGKγ activation takes place in the cytosol. In contrast, activation takes place in the membrane compartment in the wild-type model. Next, through numerical simulations, this study investigated how reducing DGKγ activation and phosphorylation rates may influence the magnitude of translocation and the membrane residence duration of PKCγ in the mutant and wild-type models. Our results show that blocking parameter k_4_ in the mutant model reduces the magnitude of PKCγ translocation, but increases membrane residence duration (Figure 7A). The results show that 80% blocking results in a 70% reduction in translocation magnitude and a near 2.5-fold increase in membrane residence time (Figure 7B). Furthermore, 90% blocking results in an 80% reduction in the magnitude of translocation and an almost threefold increase in membrane residence time of PKCγ (Figure 7A). Interestingly, the results from the wild-type model show that 80% blocking increases magnitude by fivefold and reduces membrane residence time by 33% (Figure 7B). Furthermore, 90% blocking increases magnitude by 12-fold and reduces duration by 22% (Figure 7B) compared to the baseline case of no blocking.

### How Can the PKCγ-to-DGKγ Expression Ratio Influence the Translocation Characteristics of PKCγ?

**FIGURE 8:**
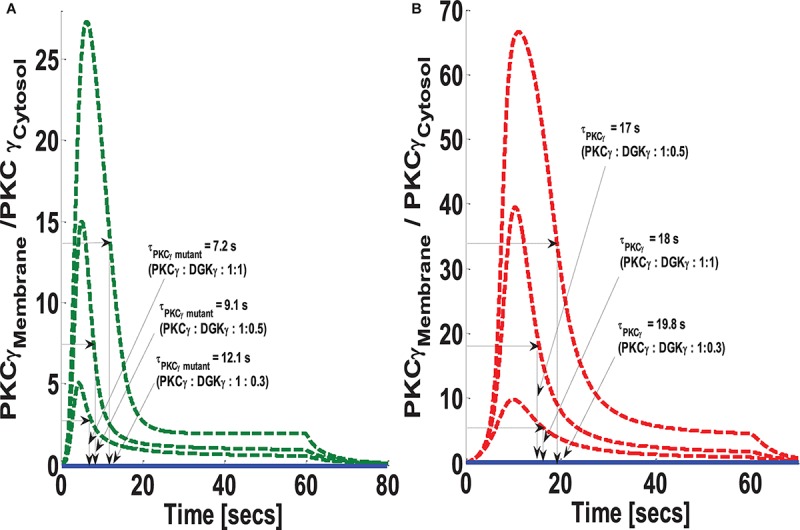
The simulations mimicking the effect of PKCγ to DGKγ expression ratio on the comparative translocation kinetics of PKCγ molecule in the mutant and wild-type models of PCs. Three different levels of expression ratios are used (PKCγ:DGKγ: 1:1; PKCγ:DGKγ: 1:0.5; PKCγ:DGKγ: 1:0.3). Here, the strength of stimulation is controlled by setting the pulse parameter “S_1_” at 20. The duration of pulse is set for 1 min; the solid line represents the non-stimulation and the dashed line represents the stimulation condition (green dashed line, mutant; red dashed line, wild-type PCs). **(A)** Translocation characteristics of PKCγ in the mutant model. Here, results indicate that reducing the PKCγ:DGKγ expression ratio increases the membrane residence time of mutant γ isoform (PKCγ:DGKγ: 1:1, τ_PKCγ mutant_ = 7.2 s; PKCγ:DGKγ: 1:0.5, τ_PKCγ mutant_ = 9.1 s; PKCγ:DGKγ: 1:0.3, τ_PKCγ mutant_ = 12.1 s). **(B)** Translocation characteristics of PKCγ in the wild-type model. Here, results indicate that reducing the PKCγ:DGKγ expression ratio first decreases and then increases the membrane residence time of γ isoform (PKCγ:DGKγ: 1:1, τ_PKCγ_ = 18 s; PKCγ:DGKγ: 1:0.5, τ_PKCγ mutant_ = 17 s; PKCγ:DGKγ: 1:0.3, τ_PKCγ mutant_ = 19.8 s).

The formulation of both models indicates that DAG levels, after stimulation, are controlled through a net negative feedback loop between its effector molecules PKCγ and DGKγ. One intriguing and still-unanswered question is how the relative expression of these molecules may influence negative feedback efficacy. In this simulation, it was assumed that both DAG effector molecules are equally expressed in the cytosol (choice of equal expressions is empirical and is chosen here to have a balanced effect on negative feedback loop). This study tested how changes in the expression ratio of PKCγ and DGKγ may influence the magnitude of PKCγ translocation, as well as its residence time, in the membrane compartment. We selected three different ratios to test. The ratios were PKCγ:DGKγ 1:1, PKCγ:DGKγ 1:0.5, and PKCγ:DGKγ 1:0.3. In both models, the systems are perturbed by a brief pulse (S_1_ = 7) which leads to rapid DAG generation in the membrane compartment. Our results show that reducing DGKγ expression in the mutant model increases the magnitude of PKCγ translocation as well as the membrane residence time (Figure 8A). It is possible that reducing DGKγ expression in the mutant model reduces the efficacy of negative feedback. Furthermore, our results indicate that reducing DGKγ expression in the wild-type models also reduces the efficacy of negative feedback. This results in increased translocation magnitude as well as increased membrane residence time of PKCγ (Figure 8B).

## Discussion

Recent evidence links mutations in the C_1_ domain of PKCγ (Yabe et al., 2003; Shimobayashi, 2016) to cerebellar neurodegeneration in SCA14 disease. It is likely that a gain of function is the mechanism causing the observed neuronal degeneration that occurs during SCA14. It is likely because the PKCγ knock-out failed to exhibit cerebellar degeneration and only showed slight ataxia (Chen et al., 1995; Kano et al., 1995). This was probably due to the loss of the pruning process at the PF-PC synapses. Evidence also suggests that mutations in the C_1_ domain could alter the activation and translocation profiles of PKCγ (Adachi et al., 2008; Shuvaev et al., 2011; Wong et al., 2018). It could be that these alterations in the enzymological properties of PKCγ and its regulator DGKγ are responsible for the dysregulation of Ca^+2^ homeostasis during SCA14 disease pathology. This disruption of Ca^+2^ equilibrium is probably due to altered gating characteristics of TRPC_3_ channels. One possible cause of TRPC_3_ gating dysregulation may be the inability of mutant molecules to completely phosphorylate TRPC_3_ channels. In this case, the Ca^+2^ influx during disease-related PCs was only partially blocked. Consequently, the intracellular accumulation of Ca^+2^ in PCs is probably an underlying cause of observed neurodegeneration in SCA14 patients (Shuvaev et al., 2011). Thus, in order to understand the mechanisms of SCA14 disease, it is essential to study the associated changes in the enzymological properties of its key molecular players (Yamaguchi et al., 2006).

This study focuses on analyzing the enzymological properties of PKCγ and DGKγ in the context of SCA14 disease. Here, through a computational approach, we compare the signaling interactions of PKCγ and DGKγ in wild-type and mutant cells. Experimental evidence shows that despite the increase in activity, the membrane residence time of PKCγ is reduced in mutant models of PCs compared to wild-type models (Adachi et al., 2008; Shuvaev et al., 2011). Interestingly, another set of observations indicate that despite constitutive activity in cytosol, the activity of mutant PKCγ is reduced in the membrane compartment (Verbeek et al., 2008). In turn, this reduction in membrane residence duration or activity of PKCγ is believed to be linked to insufficient phosphorylation of TRPC_3__._ This causes a disruption in Ca^+2^ homeostasis (Adachi et al., 2008; Henning, 2011; Shuvaev et al., 2011). Here, we investigate the key question of why membrane residence time of PKCγ decreases in SCA14 disease. This study investigates this question by proposing and numerically evaluating two contrasting signaling models of depolarization-induced DAG generation and regulation in both wild-type and mutant PCs. The models compare the possible signaling cascades of DAG both in the SCA14 mutant and in wild-type PCs through simulations.

Precise modulation of TRPC_3_ gating is essential for the viability of PCs and their characteristically dense dendritic tree (Shuvaev et al., 2011; Henning, 2011). Evidence also suggests that, in a single PC, the TRPC_3_ channels are by far the most abundant molecule compared to the other members of the TRPC family (Henning, 2011). TRPC_3_ proteins are mainly found in the PC soma and dendrites (Henning, 2011). These channels are regulated downstream of mGluR_1_ pathways and play a key role in the maintenance of calcium homeostasis in cPCs (Henning, 2011). Furthermore, experimental evidence suggests that precise gating of the TRPC_3_ channel is modulated by PKCγ in the membrane compartment (Henning, 2011; Shuvaev et al., 2011; Shimobayashi, 2016). Evidence also suggests that PKCγ may modulate phosphorylation and hence inactivation of the TRPC_3_ channel. Thus, membrane residence duration of PKCγ may play a critical role in regulating channel gating mechanisms. The membrane residence time of PKCγ is regulated by DAG, and, as DAG is converted to PA by DGKγ, membrane-bound PKC returns to the cytosol (Shuvaev et al., 2011).

The simulations in this study focused on the key question of how reduced membrane residence time of mutant PKCγ is based on comparing mutant and wild-type models under the same conditions. The wild-type model within this study is based on stimulation-induced translocation and activation of PKCγ and DGKγ. The mutant model, however, is based on constitutively active PKCγ in the cytosol (Wong et al., 2018) and activation and phosphorylation of DGKγ even during basal conditions. In the mutant model, stimulation-induced translocation of both active molecules from the cytosol to the plasma membrane leads to quick and direct metabolism of DAG in the plasma membrane compartment. Here, all the parameters in both models are set at the same numerical values and both the models are perturbed with a similar strength pulse. The goal of this is to test what happens to the membrane residence duration within two different signaling topologies. If all the kinetics, translocation, and stimulation strength parameters are kept the same, how does τ_PKCγ_ change in the wild-type and mutant models of cPCs? Here, τ_PKCγ_ is the membrane residence time of PKCγ and is defined as average residence duration during which 50% of membrane PKCγ has re-translocated back to cytosol. Interestingly, the results show that τ_PKCγ_ = 18 s for the wild-type model and τ_PKCγ_ = 7.2 s for the mutant model. Indeed, these results elucidate that alteration of topological structure between two models could lead to differences in membrane residence time. The results here indicate that PKCγ residence time in the mutant model is approximately threefold shorter than in the wild-type. These results are consistent with previous experimental recordings in PCs from cerebellar slices (τ_PKCγ_ = 19 s for wild-type cells and 6.0 s for mutant cells) (Shuvaev et al., 2011). These results support the hypothesis that constitutively active PKCγ may have shorter membrane residence duration in a mutant model of cPCs, despite higher activity levels (Adachi et al., 2008; Shuvaev et al., 2011; Wong et al., 2018).

In this study, we explore the mechanisms that might control membrane residence time of PKCγ. Our results suggest that the formation of a local signalosome in the membrane compartment is key in regulating the membrane residence time of PKCγ (Yamaguchi et al., 2006). Furthermore, our results show that, in the case of the wild-type model, the local signalosome is formed in the membrane compartment. In the case of the mutant model, the signalosome is formed in the cytosol. It seems that in the wild-type model, the depolarization-induced stimulation leads to membrane translocation of inactive PKCγ and DGKγ. Once in the membrane compartment, these molecules organize themselves into a local signalosome, which, in turn, leads to DAG metabolism and eventual disassembly of this local signaling machine. It also leads to return of both DAG effector molecules to the cytosol. Our results suggest that, in the mutant model, the signalosome between PKCγ and DGKγ is formed in the cytosol. This occurs independent of DAG and even under basal conditions.

Our results propose that, in the case of the wild-type model, the molecular processes involved in the assembly, anchoring, and disassembly of the signalosome may provide enough time for PKCγ to tightly modulate the activation, inactivation, or recruitment of its key substrates, TRPC3 channels in PCs. In contrast, our simulations suggest that, in the mutant model, signalosome is not assembled at the membrane. Instead, the PKCγ and DGKγ molecular pair interact with each other in the cytosol under basal and DAG-independent conditions. Furthermore, the results reflect that reduced residence time of PKCγ in the mutant model could be due to the lack of signalosome assembly at the membrane. This is because depolarization-induced translocation of active DGKγ molecule from the cytosol to the membrane could lead to direct metabolism of DAG into PA, without undergoing the formation of a molecular complex at the membrane. Consequently, this results in faster DAG conversion to PA and quicker return of effector molecules to the cytosol. These results support the idea that reduced membrane residence time of PKCγ is mainly linked to its constitutive activity in the cytosol (Wong et al., 2018), which, in turn, may result in differences in PKCγ and DGKγ interaction in the cytosol.

A critical assumption of this study is based on the possibility of a functional coupling between DAG effector molecules in PCs. This assumption could be disputed, as we do not have a direct evidence that such a coupling exists in PCs and is functional during the depolarization-induced translocation and activation events. We borrowed the idea of this functional interaction between DAG effector molecules from a previous experimental observation focusing on the PKCγ and DGKγ interactions in CHO cells and our previous modeling work of this system (Yamaguchi et al., 2006; Aslam and Alvi, 2019). We assumed that a similar signaling cascade could be functional in PCs. This similarity of CHO model cell and PCs could be disputed, as there may be differences in translocation dynamics of DGKγ in these cell systems. However, another set of direct observations in PCs indicate the quick degradation of DAG, cyctosol-to-membrane translocation kinetics of PKCγ, remigration dynamics of PKCγ to cytosol after purinergic receptor activation, and membrane residence duration in both mutant and wild-type models (Shuvaev et al., 2011). Based on these observations, we argue that during membrane depolarization-induced events in PCs, a coupling between DAG effector molecules could be present and functional. In the absence of a net negative feedback effect generated through PKCγ and DGKγ interactions, DAG might persist at membrane, leading to the prolonged membrane residence duration of PKCγ, which at least is not the case, as observed in some previous studies (Adachi et al., 2008; Shuvaev et al., 2011; Wong et al., 2018). In fact, the mutant PKCγ molecule is linked to shorter membrane residence duration (Adachi et al., 2008; Shuvaev et al., 2011). Additional observations in the SCA14 mutant model (Wong et al., 2018) suggest the mislocalization of PKCγ in cytosol with hyperactivity and the possibility of phosphorylating/activating its substrates in the cytosol.

Besides exploring depolarization-induced translocation characteristics of PKCγ and DGKγ molecules in mutant and wild-type models of PCs, we have also studied the influence of stimulation strength (amplitude of pulse mimicking depolarization event) on the translocation characteristics and membrane residence time of PKCγ (Figures 4A,B). Our results indicate that, in mutant models, the maximum M/C ratio of PKCγ increases fourfold with a threefold increase in stimulation strength. In contrast, the increase is 10-fold for the same increase in stimulation strength for models representing wild-type PCs. Interestingly, the membrane residence time of PKCγ in the mutant model first decreases and then increases with increasing stimulation strength. The corresponding residence and translocation characteristics of DGKγ shows decreases in membrane residence but increases in the maximum M/C ratio for this molecule with increasing stimulation strength (Supplementary Figure S1).

Furthermore, our results show that a 25- to 100-fold increase in the DAG metabolism rate leads to a 1-s reduction in membrane residence duration for the mutant and a 2-s reduction for the wild-type model. The maximum M/C ratio is reduced 10% in the mutant and 40% in the wild-type model (Figure 5). This occurs because enhancing DAG metabolism results in decreased membrane translocation and faster remigration to the cytosol. Interestingly, blocking second messenger metabolism has an opposite effect on membrane residence duration, as well as the translocation characteristics of PKCγ (Figure 6). This occurs because blocking metabolism will result in DAG accumulation at the membrane compartment, thus enhancing the membrane residence duration, as well as the M/C ratio of PKCγ. Interestingly, our results show that the effects of metabolism blocking are much more pronounced on membrane residence duration in mutant PKCγ (95% blocking of DAG metabolism results in a twofold increase in residence time) compared to the wild-type model (95% blocking results only in a 10% increase in residence time) (Figure 6). However, in the case of a maximum M/C ratio, 95% blocking of second messenger metabolism in the wild-type model results in a 5-fold increase. This increase is only 2-fold in the mutant model (Figure 6). The corresponding residence and translocation characteristics of DGKγ shows increase in the maximum M/C ratio for this molecule in the wild-type model with increase in blocking of DAG metabolism rate (Supplementary Figure S2).

Our results also indicate a role for DGKγ expression on the membrane residence duration and translocation characteristics of PKCγ (Figure 8). Recent experimental results that use the GFP-tagged PKCγ in CHKO cells indicate that the membrane residence of PKCγ could be modulated by negative feedback effects of DGKγ on the activity of PKCγ through regulating DAG metabolism (Yamaguchi et al., 2006). However, these results are based on a wild-type system. It is not understood how this negative feedback loop is affected in mutant systems when PKCγ is constitutively active. In addition, how might this influence the membrane residence time, as well as the M/C ratio of mutant PKCγ? These models assume equally proportional DGKγ expression is necessary for its negative influence on PKCγ activity. The results indicate that any changes in expression ratios of PKCγ and DGKγ will have a strong influence on the membrane residence time and M/C ratio of PKCγ and DGKγ (Figure 8 and Supplementary Figure S3). These results are consistent with previous observations (Yamaguchi et al., 2006) showing a fourfold increase in membrane residence time of PKCγ. However, the key difference between the kinetics reported in previous experimental findings and the results reported here is that the current results are based on depolarization-induced stimulation in PCs (Shuvaev et al., 2011) and experimental observations mentioned above are based on ATP-induced stimulation in CHO cells (Yamaguchi et al., 2006).

In our models, we did not attempt to simulate cytosol-to-membrane translocation and re-translocation back to the cytosol in a detailed manner (Imai et al., 2002, 2004; Luo et al., 2003a, b). This study only represented the whole complex process by introducing two first-order functions of DAG concentration. These functions describe the cytosol-to-membrane migration rates of PKCγ and DGKγ as a linear function of DAG concentration (Supplementary Material S1: Table S1 and Supplementary Material S3: Table S2). The slope of the PKCγ function is higher compared to DGKγ and adjusted to account for differential sensitivity of these molecules to DAG and calcium concentrations (Yamaguchi et al., 2006). We are not aware if there is any model proposed to describe the PKCγ and DGKγ translocation in PCs. Clearly, a complete model should account for events like DAG and IP_3_ generation, DAG binding, DAG-induced activation of TRPC3, and Ca^+2^ influx due to TRPC3 opening. In addition, there is IP_3_ diffusion into cytosol, Ca^+2^ diffusion from membrane to cytosol, Ca^+2^ release from intracellular stores, binding of Ca^+2^ with dormant PKCγ, and translocation of PKCγ and DGKγ from the cytosol to the membrane (Nishizuka, 1988, 1992, 1995; Newton, 2001, 2003; Oancea and Meyer, 1998). In addition, diffusion in the membrane occurs. However, this is beyond the scope of this study. Similarly, the models presented in this study assume that the complex process of DAG generation in response to depolarization-induced activation of the mGluR1 pathway can be simply represented by a brief pulse with certain duration and amplitude. This study has not modeled the complex processes involved in the generation of DAG and IP_3_ after the activation of the G-protein-coupled phospholipase. Rather, we have used a simplistic approach to describe these complex processes.

Some of the assumptions we made to construct these models may be disputed. This study was not able to find data points linking the effect of PKCγ mutations to the extent of its activity. Do these mutations lead to maximal activation or only partial activation of PKCγ in the cytosol? Here, we assume that mutations in PKCγ lead to maximum basal activation, which, in turn, leads to the maximum phosphorylation and activation of DGKγ in the cytosol, even during resting conditions. According to our mutant model, depolarization-induced local generation of DAG in the membrane compartment stimulates the translocation of its already-active effector molecules from the cytosol to the membrane. Active and phosphorylated DGKγ is in the membrane compartment and stimulates the DAG metabolism. This assumption may be disputed, and additional data should be generated to evaluate the DAG-binding capacity of constitutively active mutant molecule. Here, this study assumes that DAG metabolism in the mutant model is fast, and as soon DAG levels drop, both molecules relocate back to the cytosol.

Based on our wild-type and mutant models, it appears that activation and phosphorylation of DGKγ could critically influence the magnitude of translocation as well as the membrane residence duration of PKCγ in both these models (Figure 7). The blocking of DGKγ activation in the mutant model clearly shows that the magnitude of translocation and the membrane residence time of PKCγ are increased (Figure 7A). However, the same blocking in the wild-type model shows that though the translocation intensity increases, membrane residence time decreases with increase in blocking levels (Figure 7B). This is rather counterintuitive, as one would expect the increase in membrane residence duration with blocking of DGKγ activation in wild-type models too. Interestingly, as the parameter k_6_ was further blocked, we noticed that 99 and 99.95% blocking resulted in an increase not only in the magnitude of translocation but also in membrane residence duration (Supplementary Figure S4). Thus, these results indicate that reducing the activation of DGKγ reduces the efficacy of negative feedback loop.

## Conclusion

Through a computational approach, we show that enhanced PKCγ activity is linked to reduced membrane residence duration in the SCA14 mutant model. This work provides the very first simple mechanistic understanding and comparison of PKCγ temporal dynamics in wild-type and mutant models. We propose that SCA14 mutation causes the shift of PKCγ signaling from membrane compartment to cytosol, thus resulting in reduced membrane lifetime. The mechanistic nature of this work provides possibilities to increase the membrane residence duration of PKCγ through specific interventions, exquisitely targeting biochemical interactions such as blocking the DAG metabolism rate. An integrated model describing the calcium homeostasis in PCs involving DAG effector molecules and TRPC3 channels is under development and could further enhance the insights of DAG signaling in PCs.

## Disclosure

The corresponding author is part of a non-profit, i.e., BioSystOimcs, focusing on the basic R&D in drug target identification and development with no commercial plans and interests.

## Data Availability Statement

The datasets generated for this study are available on request to the corresponding author.

## Author Contributions

NA came up with the idea, carried out modeling and wrote the manuscript. FA reviewed data and helped in polishing ideas.

## Conflict of Interest

The authors declare that the research was conducted in the absence of any commercial or financial relationships that could be construed as a potential conflict of interest.
