# Supplementary material for: Simplified Model of PKCγ Signaling Dysregulation and Cytosol-to-Membrane Translocation Kinetics During Neurodegenerative Spinocerebellar Ataxia Type 14 (SCA14)
Source: Front Neurosci. 2020 Jan 31;13:1397. doi: 10.3389/fnins.2019.01397 (PMC7004970; doi:10.3389/fnins.2019.01397)
Supplement: Supplementary file 1 [file Data_Sheet_1.PDF]

Supplementary Material 1: Table 1: Numerical values of biochemical rate parameters for the mutant model of mGluR1-induced local DAG-PKCγ.mutant-DGKγ signaling in Purkinje cells of the cerebellum as described in Materials and Methods Equations (1-11):

**Parameter**	**Description**	**Numerical Values**
k_1	Kinetic rate constant for DAG generation in the PKCγ.mutant model in Purkinje cells.	1.0 sec^{-1}
k_2	Association rate constant $PKC^{A}\gamma_{II.MUTANT}$-$DGK_{II}\gamma$, binding.	0.995 $picoM^{-1}$ sec^{-1}
k_3	Dissociation rate constant of Complex C_1	0.05 sec^{-1}
k_4	Rate constant for the phosphorylation of $DGK_{II}\gamma$	0.899 sec^{-1}
k_5	Rate constant of $DGK_{II}\gamma_P$ de-phosphorylation	0.5 sec^{-1}
k_6	Binding rate constant for the DAG with $DGK_I\gamma_P$	0.95 $picoM^{-1}$ sec^{-1}
k_7	Dissociation rate constant for the complex C_2	0.1 sec^{-1}
k_8	Rate Constant of DAG Phosphrylation	0.99 sec^{-1}
k_9	Rate constant of DAGp conversion to P.A.	0.1 sec^{-1}
k_{99}	Rate Constant for DAGp dephosphorylation	0.1 sec^{-1}
λ_0	Translocation rate of $PKC^{Active}{}_{II}\gamma$-${}_{Mutant}$ from cytosol to plasma membrane	(0.06* DAG) sec^{-1}
λ_{00}	Re-translocation rate of $PKC^{Active}{}_{II}\gamma$-${}_{Mutant}$ from plasma membrane to cytosol	0.1sec^{-1}
λ_5	Translocation rate of $DGK_{II}\gamma$ from cytosol to plasma membrane	(0.006* DAG) sec^{-1}

λ_{55}	Re-translocation rate of $DGK_I\gamma$ from plasma membrane to cytosol	0.005 sec^{-1}
λ_3	Degradation rate of $PKC^{Active}_{II}\gamma$-Mutant	0.0005 sec^{-1}

Supplementary Material 2: Biochemical Reactions describing fast kinetics wild-type model: biochemical interactions describing the mGluR1-induced local DAG-PKCγ-DGKγ signaling in the wild-type model of Purkinje cells of the cerebellum. Interactions of this local molecular loop (**Fig. 2**) are based on standard Michaelis-Menten type kinetics. The following sets of biochemical reactions are used to describe the molecular interactions of this loop. The dynamic variables used are DAG to represent second messenger diacylglycerol, DGKγ to represent diacylglycerol kinase and PKCγ to represent the γ isoform of protein kinase C; a subscript I represent the concentration in first compartment which is plasma membrane and subscript II denotes the concentration in second compartment which represents cytosol; a superscript A represents activated form of the molecule; a subscript P represents the phosphorylated form of molecules. The phosphatase P is approximated as a fixed parameter. The parameter S_1 denotes the mGluR1-induced stimulation, leading to the rapid generation of DAG molecule.

$$S_1 \xrightarrow{k_1} DAG \qquad (Eq. 1)$$

$$PKC_{II}\gamma \xrightarrow{\lambda_0 = f(DAG)} PKC_I\gamma \qquad (Eq. 2)$$

$$PKC_I\gamma \xrightarrow{\lambda_{00}} PKC_{II}\gamma \qquad (Eq. 3)$$

$$DGK_{II}\gamma \xrightarrow{\lambda_5 = f\ (DAG)} DGK_I\gamma \qquad (Eq. 4)$$

$$DGK_{I}\gamma \xrightarrow{\lambda_{55}} DGK_{II}\gamma \quad \text{(Eq. 5)}$$

$$DAG + PKC_{I}\gamma \underset{k_3}{\overset{k_2}{\rightleftarrows}} PKC_{I}\gamma^{A} \quad \text{(Eq. 6)}$$

$$DGK_{I}\gamma + [PKC_{I}\gamma]^{A} \underset{k_5}{\overset{k_4}{\rightleftarrows}} C_1 \xrightarrow{k_6} [PKC_{I}\gamma]^{A} + DGK_{I}\gamma_{P} \quad \text{(Eq.7)}$$

$$DGK_{I}\gamma_{P} + P \xrightarrow{k_7} DGK_{I}\gamma + P \quad \text{(Eq. 8)}$$

$$DGK_{I}\gamma_{P} + DAG \underset{k_9}{\overset{k_8}{\rightleftarrows}} C_2 \xrightarrow{k_{10}} DGK_{I}\gamma_{P} + DAG_{P} \quad \text{(Eq.9)}$$

$$PKC_{I}\gamma^{A} \xrightarrow{\lambda_2} PKC_{II}\gamma^{A} \quad \text{(Eq.10)}$$

$$PKC_{II}\gamma^{A} \xrightarrow{\lambda 3_4} [\ \ ] \quad \text{(Eq.11)}$$

$$PKC_{II}\gamma^{A} \xrightarrow{k_0} PKC_{II}\gamma \quad \text{(Eq.12)}$$

$$DAG_{P} \xrightarrow{k11} P.A \quad \text{(Eq.13)}$$

$$DAG_{P} + P \xrightarrow{k_{12}} DAG + P \quad \text{(Eq.14)}$$

The inception and termination of the signaling in the above loop is due to local generation and removal of DAG. The signaling in the above loop starts with mGluR1-induced local generation of DAG in the lipid membrane of cPCs. This local event for the generation of a second messenger is described through equation (1). DAG generation stimulates the migration of dormant and inactive $PKC_{II}\gamma$ from cytosol to lipid membrane. This PKCγ migration event is described in equation (2). Here, the migration rate 'λ_0' is described through a function which is directly proportional to the DAG concentration (**Supplementary Material 3; Table 2**). The inactive γ-molecule in lipid membrane compartment, i.e., $PKC_I\gamma$ also re-translocate to cytosol with a fixed migration rate, 'λ_{00}' as described in equation (3). Here, the attenuator molecule of DAG signaling i.e., $DGK_{II}\gamma$ also migrates from cytosol to plasma membrane in a DAG dependent manner. This migration event is described in equation (4). The γ-molecule at plasma membrane i.e., $DGK_I\gamma$ can also re-translocate to cytosol as described in equation (5). Once at plasma membrane both $PKC_I\gamma$ and DAG bind together to form an active form of γ-isoform. This event is described in equation (6). The active molecule, i.e., $PKC_I\gamma^A$, in turn, activates $DGK_I\gamma$ through modulating its phosphorylation in the plasma membrane compartment as shown in equation (7). The active and phosphorylated DGK molecule in the plasma membrane regulates the DAG homeostasis by inducing the DAG metabolism through phosphorylation as shown in equation (9).The active form of γ-isoform i.e., $PKC_I\gamma^A$ can also migrate from plasma membrane to cytosol as described in equation (10). Here, the migration rate 'λ_3' is set as a fixed parameter. Once inside the cytosol the active form of enzyme $PKC_{II}\alpha^A$ can either follow a degradation pathway as described in equation (11) or deactivates to its dormant form as modeled in equation (12). The dephosphorylation event of $DGK_I\gamma_P$ is described by equation (8).

Supplementary Material 3: Table 2: Numerical values of biochemical rate parameters for the fast kinetics mGluR1-induced local DAG-PKCγ-DGKγ signaling in wild-type Purkinje cells of the cerebellum as described above in Supplementary Materials 2 and Equations1-14.

Parameter	Description	Numerical Values
k_1	Kinetic rate constant for DAG generation.	1.0 sec^{-1}
k_2	Association rate constant $PKC\gamma_I$ - DAG binding.	0.95 $picoM^{-1}$ sec^{-1}
k_3	Dissociation rate constant $PKC\gamma_I^A$	1.0 sec^{-1}
k_4	Association rate constant $PKC\gamma_I^A$ - $DGK_I\gamma$ binding.	0.995 $picoM^{-1}$ sec^{-1}
k_5	Dissociation rate constant C_1	0.05 sec^{-1}
k_6	Rate constant for the phosphorylation of $DGK_I\gamma$	0.899 sec^{-1}
k_0	Rate constant for the deactivation of $PKC\gamma_{II}^A$	1.0 sec^{-1}
k_7	Rate constant of $DGK_I\gamma_P$ de-phosphorylation	0.5 sec^{-1}
k_8	Association rate constant $DGK_I\gamma_P$ and DAG	0.95 $picoM^{-1}$ sec^{-1}
k_9	Dissociation rate constant for C_2	0.1 sec^{-1}
k_{10}	Rate Constant of DAG phosphorylation	0.99 sec^{-1}
k_{11}	Rate constant of DAG_P conversion to P.A	0.1 sec^{-1}
k_{12}	Rate constant of dephosphorylation of DAG_P	0.1 sec^{-1}
λ_0	Translocation rate of $PKC_{II}\gamma$ from cytosol to plasma membrane	(0.06* DAG) sec^{-1}
λ_{00}	Re-translocation rate of $PKC_I\gamma$ from plasma membrane to cytosol	0.1sec^{-1}
λ_5	Translocation rate of $DGK_{II}\gamma$ from cytosol to plasma membrane	(0.006* DAG) sec^{-1}

λ_{55}	Re-translocation rate of $DGK_I\gamma$ from plasma membrane to cytosol	0.005 sec^{-1}
λ_2	Re-translocation rate of $PKC_I\gamma^A$ from plasma membrane to cytosol	0.01sec^{-1}
λ_3	Degradation rate of $PKC_{II}\gamma^A$	0.0005 sec^{-1}

Supplementary Material 4: Supplementary Figures

Figure S_1:

The effect of stimulation strength on the comparative translocation kinetics of DGKγ isoform in the mutant & Wild type models of cPCs. Here, the strength of stimulation is controlled by setting the pulse parameter "S_1" at different levels. The parameter S_1 is set at arbitrary values of 20, 40 and 60 (inset). The duration of pulse for all these three cases is 1 minute. Here, the solid line represents the non-stimulation and dashed line represents the stimulation condition (green dashed line mutant & red dashed line Wild type cPCs). (a) Translocation characteristics of DGKγ in the mutant model. Here, results show that maximum levels of M/C ratio of DGKγ increases with increase in the strength of parameter "S_1", however, the residence time of mutant PKCγ, first decreases and then increases with pulse strength (S_1 = 20, leads to maximum M/C levels of 1 and S_1 = 40, leads to maximum M/C levels of 2.6; S_1 = 60, leads to maximum M/C levels of 8). (b) Translocation characteristics of DGKγ in the Wild type models. Here, results show that maximum levels of M/C ratio of PKCγ increases with increase in the strength of parameter "S_1", and the residence time of Wild type PKCγ, decreases with pulse strength (S_1 = 20, leads to maximum M/C levels of 1.2; S_1 = 40, leads to maximum M/C levels of 3.6; S_1 = 60, leads to maximum M/C levels of 18).

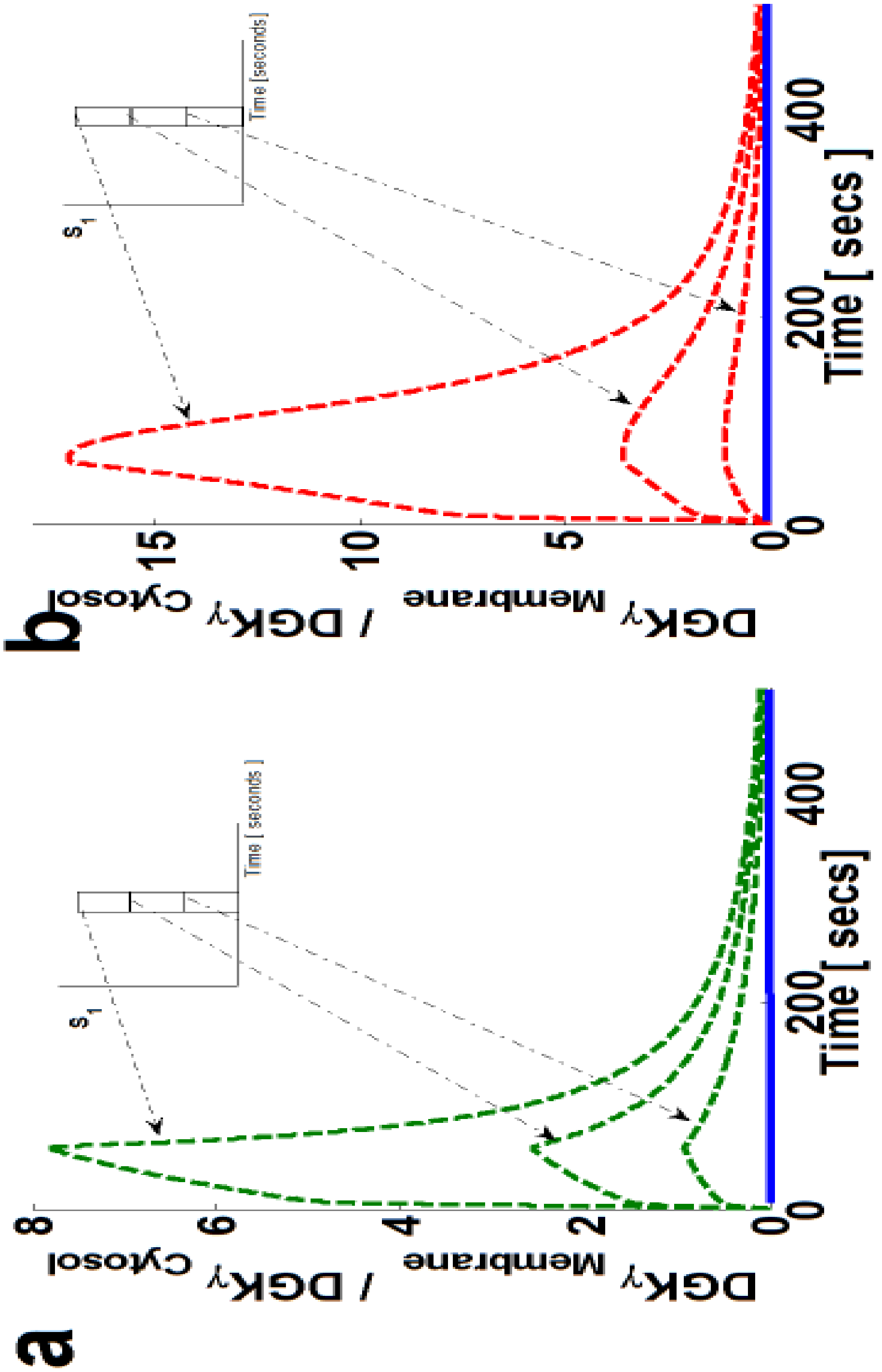
a
b
DGKγ Membrane / DGKγ Cytosol
Time [secs]
Time [secs]
0
2
4
6
8
5
10
15
200
400
s1
Time [seconds]

Figure S_1

Figure S_2:

The effect of blocking the rate constant of DGKγ phosphorylation to $DGK\gamma_P$ on the comparative translocation kinetics of DGKγ molecule in the mutant & Wild type models of cPCs. Parameter k_4 represent this rate constant in mutant models whereas, in wild types it is represented by parameter k_6. Here, the strength of stimulation is controlled by setting the pulse parameter "S_1" at 20. The duration of pulse is set for 1-minute solid line represents the non-stimulation and dashed line represents the stimulation condition (green dashed line mutant & red dashed line Wild type cPCs). (a) Translocation characteristics of DGKγ in the mutant model. Here, results show that blocking the parameter k_4, in mutant model has no effect on the translocation properties of DGKγ molecule. (b) Translocation characteristics of DGKγ in the Wild type models. Here, results show that blocking the parameter k_6, in Wild type model enhances the membrane translocation of DGKγ.

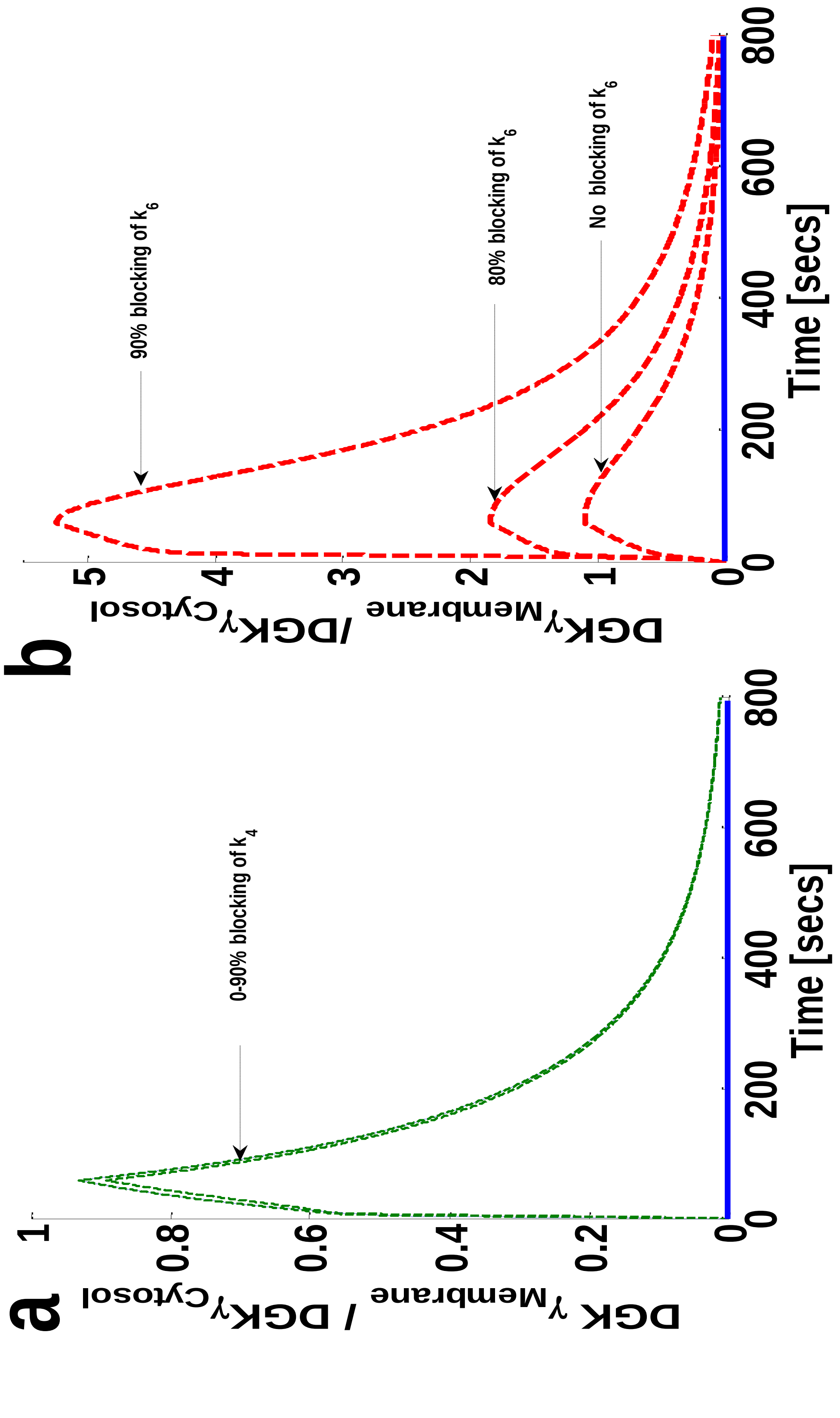

a
DGK γ Membrane / DGKγ Cytosol
0-90% blocking of k4
Time [secs]
b
DGKγ Membrane /DGKγ Cytosol
90% blocking of k6
80% blocking of k6
No blocking of k6
Time [secs]

Figure S$_2$:

Figure S_3:

The effect of PKCγ to DGKγ expression ratio on the comparative translocation kinetics of DGKγ molecule in the mutant & Wild type models of cPCs. Three different levels of expression ratios are used (PKCγ : DGKγ : 1:1; PKCγ : DGKγ : 1:0.5; PKCγ : DGKγ: 1:0.3). Here, the strength of stimulation is controlled by setting the pulse parameter "S_1" at 20. The duration of pulse is set for 1 minute, solid line represents the non-stimulation and dashed line represents the stimulation condition (green dashed line mutant & red dashed line Wild type cPCs). (a) Translocation characteristics of DGKγ in the mutant model. Here, results indicate that reducing the PKCγ : DGKγ expression ratio increases the membrane translocation of γ isoform of DGK. (b) Translocation characteristics of PKCγ in the Wild model. Here, results indicate that reducing the PKCγ : DGKγ expression ratio also enhances the M/C ratio of DGKγ isoform.

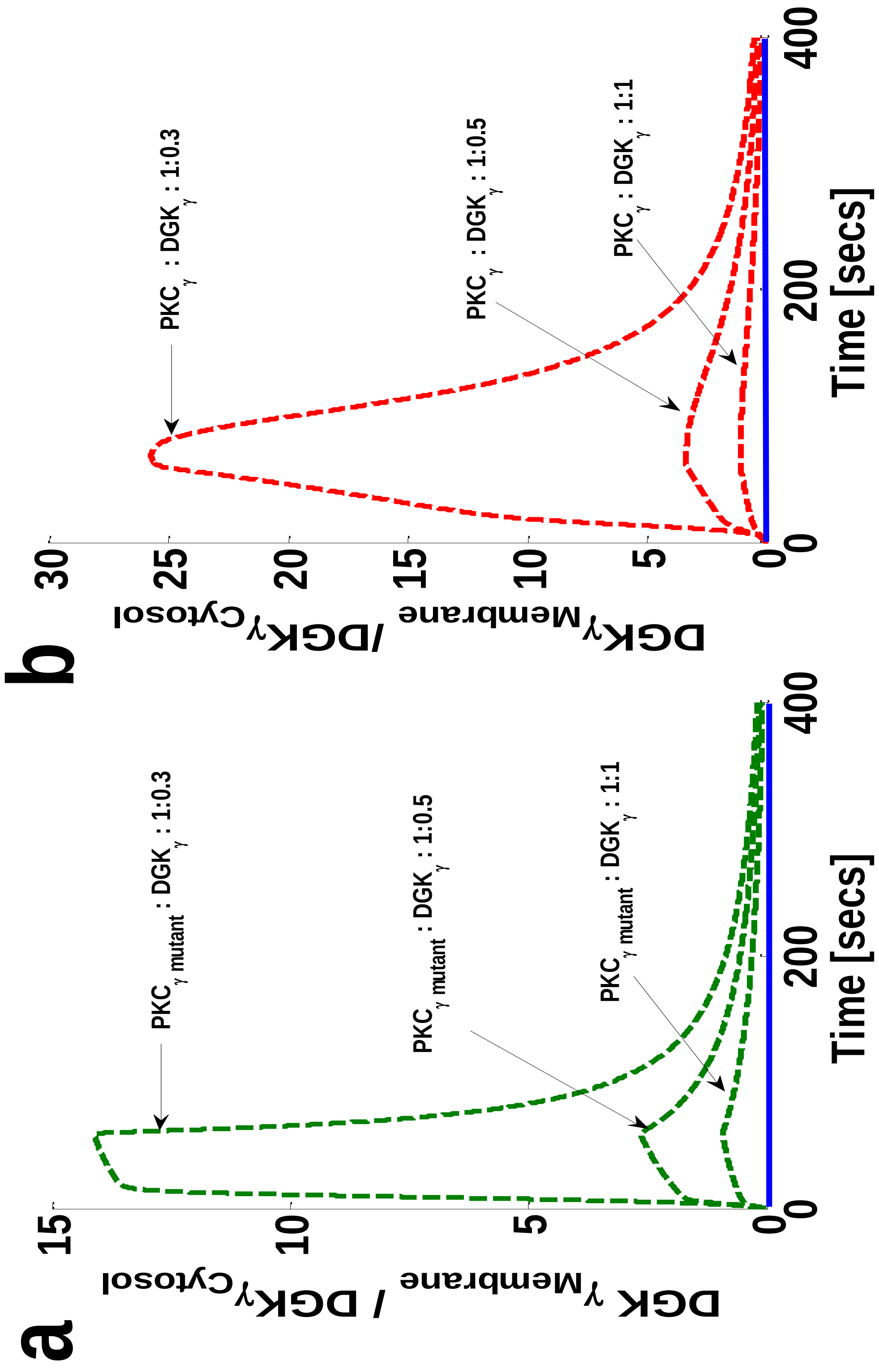
a
b
DGKγ Membrane / DGKγ Cytosol
DGKγ Membrane /DGKγ Cytosol
Time [secs]
Time [secs]
PKCγ mutant : DGKγ : 1:0.3
PKCγ mutant : DGKγ : 1:0.5
PKCγ mutant : DGKγ : 1:1
PKCγ : DGKγ : 1:0.3
PKCγ : DGKγ : 1:0.5
PKCγ : DGKγ : 1:1

Figure S3:

Figure S_4: The effect of blocking the rate constant of DGKγ phosphorylation to $DGK\gamma_P$ on the comparative translocation kinetics of PKCγ molecule in the mutant & Wild type models of cPCs. Parameter k_4 represent this rate constant in mutant models whereas, in wild types it is represented by k_6. This figure is an extension of Figure 7 in the main paper. (a) This figure shows 99% and 99.5% blocking the parameter k_4 in mutant model. (b) This figure shows 99% and 99.5% blocking of parameter k_6 in the Wild type model.

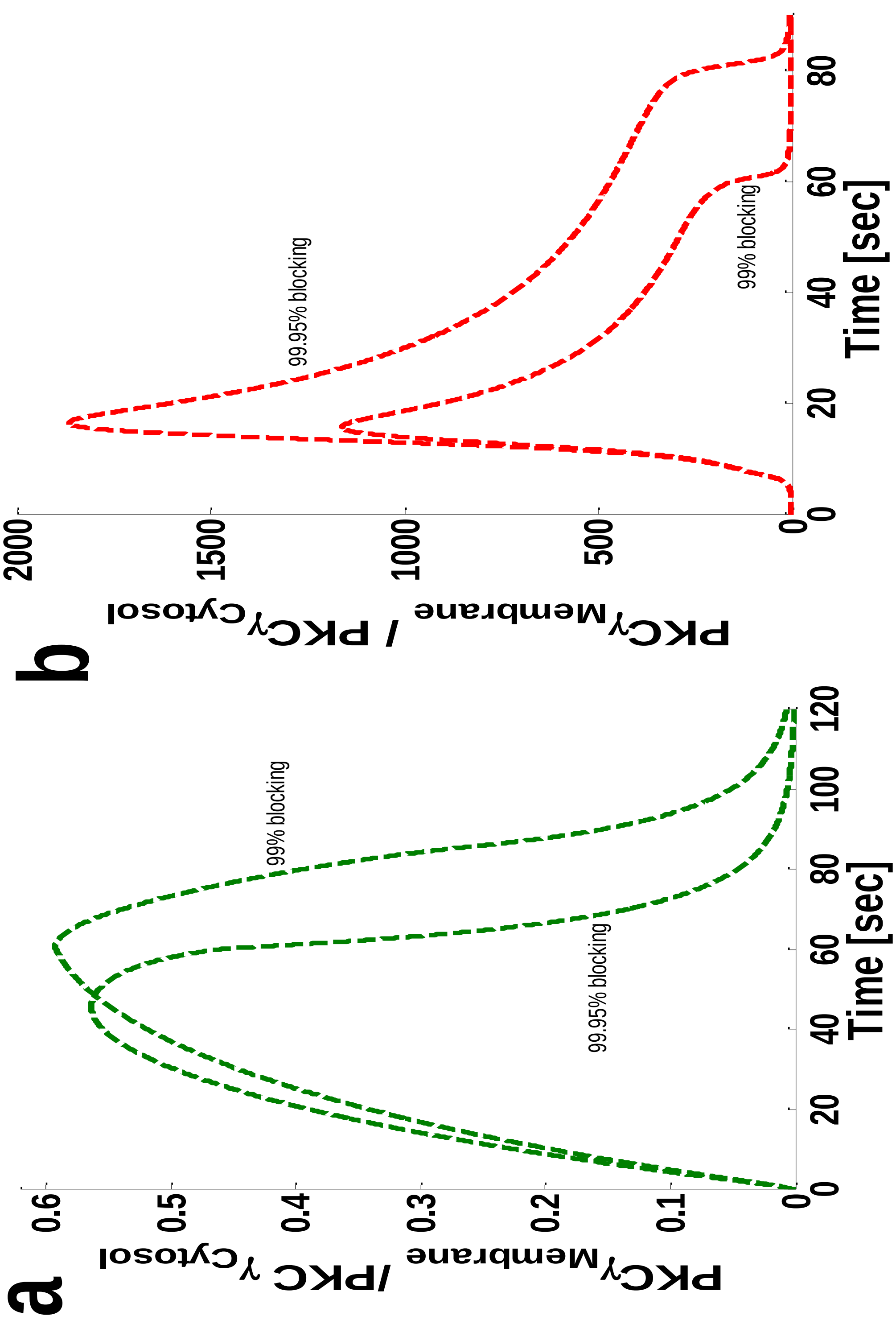
a
b
PKCγMembrane /PKC γCytosol
PKCγMembrane / PKCγCytosol
Time [sec]
Time [sec]
99% blocking
99.95% blocking
99.95% blocking
99% blocking

Figure S4:
